# Frontline learning of medical teaching: “you pick up as you go through work and practice”

**DOI:** 10.1186/s12909-017-1011-3

**Published:** 2017-09-19

**Authors:** W. Hartford, L. Nimmon, T. Stenfors

**Affiliations:** 1 Centre for Health Education Scholarship, University of British Columbia, Vancouver, V6T 1Z4 Canada; 2 Department of Occupational Science and Occupational Therapy, Faculty of Medicine, University of British Columbia, Vancouver, Canada; 3 Department of Learning, Informatics, Management and Ethics, Karolinska Institutet, Stockholm, Sweden

**Keywords:** Physician, Faculty development, Qualitative research, Feedback, Peer observation

## Abstract

**Background:**

Few medical teachers have received formal teaching education. Along with individual and organizational barriers to participation in teacher training programs, increasing numbers and altered distribution of physicians away from major teaching centers have increased the difficulty of attendance. Furthermore, it is not known if traditional faculty development formats are the optimal learning options given findings from existing studies document both positive and negative outcomes. There is a gap in research that explores *how* medical teachers learn to teach and also limited research regarding *how* medical teachers actually teach.

The purpose of this study was to provide insight into how physicians describe their teaching of trainees, and the nature of their teaching development and improvement to inform faculty development programs.

**Methods:**

Semi-structured interviews were conducted with 36 physicians, with a broad range of teaching experience, purposefully selected from five disciplines: Internal Medicine, Pediatrics, Psychiatry, Surgery, and Family Medicine. A qualitative, inductive approach was used to analyse the data.

**Results:**

Teaching was described as being centered on the needs of individual trainees, but was dependent on patient presentation and environmental context. For this group of physicians learning to teach was perceived as a dynamic and evolving process influenced by multiple life experiences. The physicians had not learnt to teach through formal education and then put that learning into practice, but had learnt to teach and improve their teaching through their trial and errors teaching. Life experiences unconnected with the medical environment contributed to their knowledge of teaching along with limited formal learning to teach experiences. Teaching practice was influenced by peers and trainees, feedback, and observation. The findings suggest these medical teachers learn to teach along a continuum largely through their teaching practice.

**Conclusion:**

The findings suggested that the participants’ major resource for learning how to teach was informal experiential learning, both in and out of the workplace. This may have implications for faculty development strategies for medical teaching education.

## Background

Few medical teachers have received formal teaching education [1–3] despite the recognition of teaching as a core professional attribute [2, 4]. Traditionally, faculty development units associated with universities offer training provided in a variety of formats such as workshops, short courses, university accredited awards, and on-line webinars etc. [1–3, 5–7]. However, the difficulty of encouraging physician attendance has been increased with the higher number and altered distribution of medical teachers away from major teaching centers [8–10]. In addition, there are several other barriers, both individual and organizational in nature, for physician participation in faculty development programs some of which are lack of protected time, underestimation of the need for further training, and lack of personal motivation [5, 11, 12]. Furthermore, it is not known if traditional faculty development formats are the optimal learning options as learning also occurs in informal real life contexts [2, 3, 6, 7, 13], and through social mechanisms such as hidden curricula [5, 13].

Few studies have assessed the effect of intensive teacher training on the performance of medical teachers [2, 3, 5]. Findings from existing studies are mixed, suggesting both positive outcomes: improved teaching ability [12, 14], and negative outcomes: decreased teaching effectiveness [13]. The field of medical education encourages self-directed and life-long learning [5, 7, 15, 16]. Being part of the medical culture medical teachers may, themselves, consider continuous development and improvement essential [12]. Although the importance of professional development for continuous development and maintenance of competency is well established there is a gap in research that explores *how* medical teachers perceive their teaching and *learning* to teach. Filling this gap may provide insight into the nature of physicians’ teaching development [2, 17].

## Methods

For this research we explored how physicians 1) describe the process of teaching trainees, and 2) describe the process of learning to teach and how they would go about becoming better teachers of trainees. Five disciplines which represent the top five selections of Canadian medical graduates and top five available Canadian Resident Matching System (CaRMS) [18] match residencies, were selected for this study for their significant representation of medical teachers and trainees, and potential for teaching opportunities. Four were Royal College specialties: Internal Medicine, Pediatrics, Psychiatry, and Surgery. These disciplines also provide a variety of inpatient and outpatient care. The fifth discipline family medicine, while not a Royal College specialty, is conceived using a similar framework to the Royal College of Physicians and Surgeons of Canada [19]. We used a qualitative approach and conducted semi-structured interviews with 36 physicians. Ethical approval from the University of British Columbia’s Behavioural Research Ethics Board was obtained for the study. This research was part of a broader exploratory study that was designed to investigate physicians’ conceptions of their teaching practices and their development as teachers of trainees as well as their conceptions of teaching and learning to teach their patients [20, 21], and their understanding and application to teaching trainees of the Canadian Medical Educational Directives for Specialities (CanMEDS) framework. In addition, the investigation explored how physicians perceived power relations within their patient and trainee relationships which has been reported on elsewhere [22]. Approximately 40% of the interview questions pertained to physician interactions with trainees. For example: Can you describe to me your approach to teaching different trainees in order to teach them well? and, Can you tell me about how your teaching of trainees has evolved over time?

### Context, population, sample, and data collection

This study took place at the University of British Columbia. The Faculty of Medicine teaches undergraduate medical students and postgraduate residents at a main campus and three regional campuses. All clinicians who teach medical students and postgraduate residents hold a faculty appointment. Academic clinicians are salaried with formal expectations and protection for research and teaching; clinical faculty are funded for sessions in which they teach while they deliver patient care. Participants in this study were drawn from university-affiliated academic health sciences centers, community hospitals, and clinics in the main site. We used purposeful sampling to provide us with physicians who collectively represented a broad range of teaching experience. An email introductory letter that described the larger study was sent to all colleagues identified by the study’s co-investigators. On average 4 out of 6 of those physicians initially contacted in each discipline were interested in participating, and were subsequently sent a consent form prior to the interview taking place. To recruit the additional participants we used a snowball sampling technique which involved asking those interviewed to provide names and email addresses of colleagues who fit our recruitment criteria. An introductory email describing the study was sent to the additional potential participants. The interviews were held either in person at the participant’s office or on the phone and averaged 1 h in duration. Participants received a gift certificate.

The interviews were conducted by WH and LN (authors) with six interviews conducted by a Family Medicine research associate. All interviews were audio recorded, transcribed and given a unique identifier based on speciality and number: Family medicine is identified FAM, Internal medicine as IM, Pediatrics as PED, Psychiatry as PSYCH, and Surgery as SURG. After interviews had been conducted in three specialties all transcripts were read and LN and TS reviewed and revised the initial interview protocol which was based on two pilot interviews. Since the purpose of the interviews was to explore aspects of the learning process in respect to teaching trainees, most questions were followed by probing and follow-up questions to deepen the respondents reflexive thinking about learning [23].

### Data analysis

The audio recordings of the interviews were transcribed verbatim and the transcripts were analysed by WH, LN, and TS using QSR NVivo Version 10 (QSR International Pty Ltd., Doncaster, Victoria, Australia). The inductive analysis approach consisted of organizing the data, immersion in the data, generating categories and themes, and coding the data using a category system coding scheme [23]. The initial data organization was guided by the research questions: 1) How do medical teachers describe teaching trainees? and 2) How do medical teachers describe learning to teach and becoming better teachers of trainees? As analysis progressed, categories and themes were identified and discussed by the research team. During the final stage of analysis, TS and WH brought the several themes together to create an overall structure of the main patterns.

We used investigator triangulation to strengthen the quality of the study. The mixed backgrounds of the investigators, medical education (TS), adult education (WH) and social science (LN), provide suitable and complimentary lenses through which to analyze the data, and may increase trustworthiness by lessening the probability of common unified assumptions that can occur within a small research team [24].

## Results

The findings reflect participants’ descriptions of how they taught trainees, and how they learnt to teach. This group of physicians approached teaching trainees in various ways. In general, teaching was reported to be centered on the needs of individual trainees, but at the same time was dependent on the patient presentation and environmental context. For this group of physicians learning to teach was described as a dynamic and evolving process influenced by multiple life experiences: a continuum of learning. The physicians reported learning to teach and improving their teaching through their practice of teaching. Life experiences unconnected with the medical environment were perceived as contributing to their knowledge of teaching along with limited formal learning to teach experiences. Interactions with peers and trainees were also cited as being influential to their teaching practice. Rather than present the findings separated according to our two research questions we have merged the findings to acknowledge the continuum of learning [1] to teach through teaching.

We present the findings as two themes: 1) Physicians, trainees, context, and teaching, and 2) Respondents’ experiences of learning to teach.

### Physicians, trainees, context, and teaching

In this section we describe the physicians, their trainees, their sites of teaching, and their teaching.

### The physicians

In total 36 physicians were interviewed from five specialties: 12 FAM (7 male, 5 female), 6 IM (1 male, 5 female), 6 PED (2 male, 4 female), 6 PSYCH (3 male, 3 female) and 6 SURG (6 male). The length of time in clinical practice ranged from 5 to 41 years with an average of 19 years, and the average length of time teaching trainees was approximately 16 years.

Not all physicians had wanted to teach and the participants identified various reasons for taking on a teaching role such as familiarity with the program and:

*“There wasn’t really anybody else to teach”* (F3 FAM).

*“I didn’t want to teach. I didn’t think that was me at all. But after getting into it, we teach every day. And I like it a lot more than I thought I would*” (F2 FAM).

There were indications that these physicians perceived teaching trainees as a necessary part of their work. Positive teaching experiences re-enforced their motivation to teach. These medical teachers identified and shared a variety of motivators for teaching such as: giving back to the medical community, watching trainees grow in knowledge and confidence, seeing trainees become good clinicians and colleagues, passing on knowledge and experience to the next generation to ensure better patient care, learning from learners, social interactions and professional collaboration with trainees, capturing the interest of trainee either in their specialty or approach to practice, and the youthfulness and enthusiasm of trainees.

*“I think I really enjoy watching residents and students grow their knowledge and confidence and just become really good clinicians”* (F1 FAM).

*“…it’s about making sure that the trainees become good quality physicians that end up taking care of patients*” (M3 IM)*.*

Approximately one third of the participants described their active involvement in research.

*“But the research hasn’t been in terms of training. It’s been more clinical research*” (H1 PSYCH).

### The trainees

The majority of physicians taught a range of trainees from 1st year medical students to residents. Some physicians also taught fellows and acted as supervisors for doctoral students. In most instances physicians received one or two trainees at a time with the duration of placement depending on the level of trainee and the program requirements. Physicians also taught trainees from different specialties. For instance, F6 FAM taught a mix of family practice and psychiatry 1st and 2nd year residents, and H4 SURG taught medical students through to fellows from a variety of specialties as well as other health professionals such as nurses. Several physicians variously taught seminars to groups of trainees, and taught around trainees learning goals during residency and career paths.

### Context

All of the medical teachers reported that most of their teaching occurred in clinical settings such as rounding on patients, and consultations at the hospital or office depending on where they practiced. The study participants described teaching trainees as being context and environment dependent, case based, and patient-orientated which was identified by one physician as:

“…*learning off [sic] what walks through the door”* (F3 FAM), and as:

“…*is dependent on what they see and that is unpredictable. It’s not like we have a prescriptive patient load every three months to meet their teaching needs in a perfect way”* (H2 SURG).

Several physicians indicated that their practice was fee-for-service. Teaching trainees reduced the number of patient consultations, reduced income, increased costs, such as operating room expenses, and had an impact on consultation and medical procedure wait lists.

“*Financial return. Because it takes a lot of time. And sometimes-- taking on too my residents for sure will slow down our practice. And then we suffer financially”* (F6 FAM).

Physicians described balancing trainee education while managing busy practices and ensuring patient care was not adversely affected. Although patient consultations were reduced, physicians perceived the time with trainees was less than optimal.

“*The balance of education and service in our teaching hospital is always an ongoing struggle. And if there’s just simply too much to do for patients in a set number of time”* (F3 PED).

### Physicians describe their teaching of trainees

*“And you have to be able to be looking for, in every encounter you have with a patient where a trainee’s involved, as to how can I provide value to the trainee in this encounter”* (H3 SURG)*.*

The majority of medical teachers identified using “a little bit of everything” (H2 PSYCH), and variously described a variety of formal and informal teaching methods, didactic teaching, lecturing, homework, and discussions and conversations. Some physicians indicated that they did not teach through lecture and seminars as much as through informal forms of teaching that arose during clinical practice.

*“…it’s quite informal…there are different places I teach, so I’ll teach them on the ward, at the bedside. I think that’s the best learning experiential…”* (F2 PED).

More than half of the physicians described their teaching as evidence based, referring to medical/clinical scientific evidence for what they taught rather than how they taught. Virtually all participants indicated, either explicitly or implicitly, that they tailored their teaching to the learning objectives and goals of trainees. Tailoring was characterised as requiring an understanding of the trainee’s background, considering different levels of competency, grading responsibility, and providing increasing autonomy with increasing learner ability.

*“They will then observe, and then I will-- then they will do it, I will guide them, and then they’ll do it all by themselves [sic] with me watching. And we’ll recap on it later”* (M6 FAM).

A role modeling approach to teaching trainees was described by almost two thirds of the participants particularly in relation to integrating learning around non-medical skills:

*“…along the way, we talk about…we talk about the other skills of being a physician”* (H1 PSYCH).

In order to teach well, several of the medical teachers reported using various tools for teaching such as relevant literature, educational material, websites, and diagrams. Others explicitly referred to providing timely feedback as part of their approach to teaching trainees. The majority of medical teachers referred to the importance of creating learning environments in which trainees felt comfortable and safe to answer questions.

*“So I think there has to be that environment of learning and warmth and nurturing where people can feel quite safe”* (F2 PED).

All of the physicians perceived that lack of time to teach adversely impacted their teaching of trainees.

“*I think again sometimes we just have so many patients and it’s so busy clinically that you really just don’t have the time to sort of sit down for half an hour and chat about this particular topic. So I think time is a limiting factor*” (F1 PEDS).

### Respondents’ experiences of learning to teach

Our analysis identified four distinct processes and experiences described by the respondents regarding how they learned to teach and improve their teaching practices: learning through experience, formal training, receiving feedback, and peer observation.

### Learning through experience

These physicians explained how they learned to teach based on three types of experiences: from outside of clinical practice medicine, from being a trainee or a student themselves, and from teaching experience in their role as physicians.

### Learning through experiences outside clinical practice

*“I think life experience changes the way you teach. So when I first started off I was married but without children. Now I have three kids. So I think having kids-- a lot of my residents learn about children through my kids. Or just through my stories about my kids…sleep training, feeding, behavioural stuff…that’s when you bring in your life piece into teaching”* (F1 FAM)*.*

Several of the physicians reported that their motivation and ability to teach had been influenced in some way by earlier life experiences. For example, life experiences outside of medical practice which had influenced teaching practice included experience as a girl-guide leader, an English as a second language teacher, a life-guard instructor, having parents who were teachers, and becoming a parent. Three physicians referred to drawing on their experiences teaching in other parts of the world and two participants cited involvement with governance bodies such as the Royal College and teaching committees as influencing their teaching practice.

### Learning through experiences as a trainee

*“And I recall once…being mauled by an unpleasant senior physician when I was a student. And I vowed I’d never do that to anyone myself”* (M6 FAM).

Several physicians spoke about their personal negative training experiences which had influenced their teaching practice. Another physician reflected that she had learned as a trainee from less credible teachers that she needed to teach in an “evidenced based manner” if she was to be credible and earn her trainees’ respect. More positively, several physicians referred to learning to teach from supervisors who had spent time helping them.

### Learning through experiences as a teacher

Several physicians referred to learning to teach through the experience of teaching other trainees during their own training.

*“To some degree you learn it in school, you know, to some degree you learn it through supervisors …But most of it, I think, you pick up as you go through work and practice”* (M4 PSYCH).

A principal theme that emerged from our data was that of learning through the practise of teaching trainees. The physicians variously reflected that experience and practice brings expertise, wisdom, calmness and a greater confidence in their teaching skills and knowledge, and a deeper understanding of the characteristics associated with being a good teacher. These characteristics included acquiring good communication skills, and the ability to engage learners, being honest with feedback, building trust and patience, and having the ability to provide trainee learning opportunities.

Experience was also reported as bringing an awareness of self, teaching skills and approaches to teaching. Many of the physicians described their learning of good teaching skills as an evolutionary and lifelong learning process through the experience of teaching trainees.

*“So I don’t think you can ever say you will always have one teaching style or you shouldn’t. I think in order to evolve your teaching will evolve”* (M3 IM)*.*

Physicians variously identified how teaching experience had enabled them to become what they perceived as better teachers. For example, teaching experience led to feeling less pressured “to teach everything at once” (F5 IM), development of a teaching style and personality, becoming more learner centric, tailoring learning to trainees needs, and practising more evidence based teaching, and moving from didactic teaching to more case based teaching.

A few physicians suggested that self-reflection on teaching practices provided motivation to become a better teacher and required admitting both to self and trainees the limits of their knowledge, acknowledging strong and weak teaching points, and identifying what was not working*.*

*“So I had a lot of time to think and reflect on it and had the same groups over and over again, like, the same subject groups but with different students. So I would just try different things and try to figure out what worked.”* (H1 IM)*.*

Other physicians alluded to honing skills, fine tuning information, and/or being “up-to-date” as ways to improve teaching.

### Formal training

*“I didn’t know how to teach. They didn’t tell us how to teach…So it was a few years after that that I started going to the train the trainers-- the teacher’s toolbox. And I thought, oh, my goodness, there’s way more to it than I thought there was… I think I’m a much better teacher than I used to be ‘…cause I know more about what I’m supposed to do”* (F2 FAM)*.*

The majority of physicians had received little or no formal teaching education or basic training courses prior to their first trainee teaching experience, and some had no basic training even after many years of teaching. A few of the physicians had received some training teaching other students or residents as part of their overall medical training. Several of the physicians had engaged in learning to teach opportunities provided by various institutions such as the Canadian Pediatric Society, and university courses. Opportunities included formal workshops, courses such as the One Minute Teacher, ABC Primer and Train-the-Trainer, formal courses, and undertaking a fellowship. Bringing in international speakers to give rounds and workshops was identified by one respondent as another method used to improve teaching practice. A perceived benefit of taking courses was improved focus on teaching. Several physicians identified conferences, and courses on adult learning theory, stages of learning, and acquiring teaching skills as opportunities for continued learning.

“…*if I get an opportunity I will go to any workshop that will improve my teaching skills”* (M3 PSYCH).

Several of the medical teachers identified, and had sought out opportunities for improving their teaching skills and knowledge base; such as, learning how to improve their communication skills, their ability to provide constructive criticism and feedback, and how to encourage critical thinking.

*“It’s like I realize I have a hard time giving negative feedback so if I’m going to do better, I have to work on improving those skills.”* (F1 IM).

To improve teaching efficacy, a few physicians identified organising acquired knowledge into structured teaching practices as well as “setting up a more solid infrastructure” (M4 PSYCH) for trainees. Two physicians identified a need for more teaching related faculty development courses, and other physician suggested discussion groups with a panel of excellent medical teachers would provide opportunities for improving teaching skills.

*“I think I would like to probably work on sort of having a more organized approach to sort of, you know, teaching around particular topics”* (F1 PED).

However, although many of the physicians knew about various teaching courses, some physicians indicated that they would not engage in learning to teach opportunities citing lack of motivation, no perceived need to learn more about how to teach, and time constraints as contributing factors.

### Learning about teaching through the process of receiving feedback

*“So if you were to ask me right now what specific skill set I was short of, I would have to say I don’t know. But that’s not because I know all the skill sets. It’s probably just ‘cause there’s some things that I’m not aware of”* (F8 FAM)*.*

Respondents described how receiving feedback from both trainees and peers identified teaching skills that could be improved which helped them to improve their teaching and develop as teachers.

### Learning through receiving feedback from trainees

*“Yeah, and I also sort of try and get feedback from residents and students on how to improve things. Because they are the one who sort of are really judging me, observing me. So they are the best critics, right”* (F4 PED).

More than a third of participants indicated that receiving feedback from trainees had influenced their teaching practice in a variety of ways such as by inspiring change, confirming good teaching, and enabling medical teachers to develop programs that would better suit the needs of the trainees.

*“What I would like is ongoing, more robust feedback from trainees about the strengths and the weaknesses”* (H2 SURG).

One third of the medical teachers cited that receiving feedback from trainees which identified strengths and weaknesses, and areas for improvement would inspire them to improve their teaching. However, these physicians perceived current evaluation systems as inadequate and that a better evaluation system, that provided frequent, timely, and helpful feedback which identified a teacher’s weaknesses, and how teachers could better serve trainees’ learning needs would assist medical teachers in becoming better teachers. Some of the physicians perceived the need for more time to schedule feedback sessions with trainees and an environment that was conducive to providing timely feedback. However, several participants suggested that fear of recrimination may prevent or made it difficult for trainees to give negative feedback which potentially rendered evaluation reports unhelpful.

### Learning through receiving feedback from peers

*“…we do periodic assessment from an outside observer of teaching, through the university. So I have that periodically”* (H6 SURG)*.*

While only two physicians referred to having received feedback from their peers several other physicians indicated they would be receptive to the practice of peer evaluation. These physicians considered that receiving peer feedback and evaluation would make a significant contribution to teaching improvement by identifying weaknesses and should not be avoided.

*“And I should probably get a peer to supervise my teaching and to give feedback”* (H2 PSYCH)*.*

However, only one physician identified a formal peer feedback and evaluation mechanism, and another referred to the absence of such a mechanism. One physician commented that despite the desire for feedback and evaluation in practice this may not occur as physicians often worked in autonomous silos.

### Learning from peer observation

*“I think it always helps to see how other people teach, so I think I would spend time with some of my colleagues to see what their approach to teaching is and what they allow people to do and how that relationship works…”* (H3 SURG).

Various participants described how peer observation had influenced their teaching practice. These medical teachers described how informal observation of colleagues and excellent teachers interacting with trainees, and observing and listening to more experienced teachers with different teaching methods had provided learning experiences. Another physician considered that to develop as good teachers physicians required early exposure to good teachers with good communication skills who had the ability to provide constructive criticism to learners. Notably, however, two physicians suggested that observation of poor teaching skills had also served as a learning model.

*“And frankly, if we’re good or bad at it, they’re learning one or the other. So, you know, and when I was a trainee, when I saw people who are poor collaborators, I learned just as much from that experience as I did from a good collaborator”* (H2 SURG).

Although peer observation was valued as a learning experience, difficulties with this practice were identified by two participants since “we work in silos” (F2 FAM).

## Discussion

Despite the differences in these medical teachers’ characteristics, their descriptions of teaching trainings and of learning to teach were remarkably similar. The sample size was too small to draw any conclusion regarding differences between specialties. The data also suggests that sites of practice played a significant, and potentially preferred role in these participants’ informal teaching and learning to teach activities. Although these medical teachers reported limited formal teaching training, they described competent teaching practices, despite teaching in environments which they perceived to be less than optimal for learning.

### Physicians describe their teaching practice

The Canadian Medical Education Directives for Specialists (CanMEDS), a competency framework that has been adopted internationally, identifies teaching as a significant part of the Scholar role with a number of competencies that medical teachers are expected to achieve [1, 4]. Our descriptive data suggests that the physicians in this study may practice many of these competencies such as the use of formal and informal curricula, learner assessment, needs assessment, optimization of the learning environment, role modeling, providing feedback, supervising and grading responsibility, the use of evidence based techniques, and the need to tailor teaching to trainees’ requirements. Srinivasan et al. [1] present a teaching competency framework with six core competencies which also emphasises the acquisition of these same skills as desirable for a medical teacher.

### Learning from experience

The physicians in this study mostly considered they lacked formal teaching training and described learning to teach predominantly through their lived experience of teaching trainees. This finding is congruent with literature concerning formal learning to teach education for physicians [1–3], and workplace and experiential learning which have been identified as significant and valuable approaches for providing learning opportunities [2, 3, 6, 7, 25, 26]. Workplace learning provides a contextual and authentic experience as medical teachers can learn from each other through the practical application of the programs they teach [6, 7, 25, 27], and may have greater relevance to learners than formal programs and text book learning [27].

These medical teachers described learning to teach as an ongoing interactive process which was influenced by a multitude of life experiences outside of medical practice and through learning in the roles of both a trainee and medical teacher. The concept of learning to teach trainees as an ongoing process is reflected in the literature which suggests that learner physicians are influenced by both positive and negative learning experiences throughout time such as specific lectures, particular bedside teaching moments, behavior of their preceptors, and relationships [3, 5, 6, 26].

Learning from experience is a reflective process [28]. However, although our participants appeared to be quite reflective about learning to teach few of our participants explicitly described engaging in reflective practices on their life and work experiences.

### Learning from formal learning experiences

In our study participants who engaged in formal faculty development programs identified their experiences as beneficial. This supports the literature which suggests that participants are usually satisfied with programs, find the content relevant and useful, gain in knowledge and skills, and experience positive changes in attitude toward teaching [7, 11, 12, 17, 26, 29]. However, although research concerning the effects of faculty development programs on teaching and student learning is limited [2, 3, 5, 13], traditional formats may not bring about teaching improvement, may not be engaging, and may be disassociated with reality [14]. Not all of the study physicians were motivated to participate in faculty development programs citing several perceived barriers such as lack of time and no need to learn more about teaching, which also reflects what has been identified in the literature [5, 11, 12, 29]. That only two physicians identified a need for more medical teacher training courses was perhaps a reflection of perceived barriers, or possibly lack of self-awareness of the need to improve teaching skills which is considered a significant barrier to learning [11].

### Learning from feedback

As has been observed in the literature [3], receiving feedback from both trainees and peers was highly valued by this group of physicians. Also in accordance with the literature [7, 30–33], the frequency and reliability of feedback received from trainees was reported to be inadequate and although many physicians were receptive to feedback, peer evaluation and feedback seldom occurred. Several physicians indicated that the environment in which they taught was not always conducive to receiving timely and reliable feedback: since trainees may fear recrimination; and because physicians often work alone and are not in an environment that is favorable to receiving peer evaluations. Feedback may lead to reflection on practice which is considered an important component of learning [2, 6, 7, 25, 31, 34–37].

### Learning from peer observations

Several of the physicians either explicitly or implicitly referred to learning from observing teaching practices of their colleagues and teachers even when the teacher may not show expertise in a particular professional behavior, such as collaboration. Peer observation is considered necessary to physician learning and self-improvement [2, 3, 5, 7, 16, 32–35, 38], but learners need to differentiate between positive and negative behavior traits [17, 35, 36, 38].

### Obstructions and influences for teaching and learning to teach

The last, but not insignificant finding refers to organizational structures which inhibited teaching of trainees and the influences which motivated these medical teachers to continue to teach despite various obstructions. While adequate remuneration, particularly for the medical teachers practicing within the fee-for-service structure, may allow for scheduling adequate time for teaching trainees, the potential for negative impact on patients such as increased wait times for consults and/or procedures may remain. Several authors have suggested that it is necessary to identify and address organizational practices and structures which promote or impede successful workplace experiential learning [2, 3, 5, 7, 12, 14, 25, 34]; for example, the challenge of working in isolation [5]. Our findings suggested that organizational practices such as intermittent and unhelpful trainee feedback, perceptions of working in isolation, and lack of peer evaluation may not organically generate informal learning in the workplace. As with previous studies [2, 3] the medical teachers in this study appeared to derive personal satisfaction from teaching trainees which motivated them to teach. In addition, it can be inferred from the data that for these physicians teaching was, to some degree, an inherent part of their medical practice.

### Implications for practice

Learning from experience and practice in the workplace has a major role in medical teacher development [2, 3, 6, 7, 25, 26]. However, workplace learning appears to be an under-utilized resource for faculty development programs [6]. Informal learning, like formal learning, can be intentional and planned [2, 25, 34] and it is feasible that informal faculty development programs could be implemented in the workplace. It is likely that medical teachers’ learning needs and organizational structures vary. For instance in our study many, but not all, of the medical teachers practiced within a fee-for-service structure. Therefore, it may be necessary to tailor faculty development programs to fit the requirements of specific teaching and learning communities. Finally, teaching skills and knowledge acquired through formal learning programs may not easily be transferred in a stable way to the real teaching environment [3, 6, 13, 14, 25, 39]. Situated cognition theory proposes that doing and knowing cannot be separated, and that the context and culture within which an activity occurs constructs and re-constructs knowledge [39, 40]. Accordingly, learning in situ may support acquisition of knowledge and provide medical teachers with enriching practical learning opportunities [39, 40], such as regular peer observation, evaluation, and feedback, [2, 3, 6, 7, 14, 17, 32], which may help physicians develop a range of teaching skills.

### Strengths and limitations

The findings of this study provide new insight into medical teachers’ perceptions of their approaches to teaching, and of their experiences of learning to teach. However, the respondents’ descriptions of their teaching practice were not supported by field observations and may not reflect their actual teaching practice. This is a single centre study limited to medical teaching in Canada. In addition, participants only represent the five most frequently chosen medical specialties and their respective teaching cultures.

### Suggestions for further research

We identified four areas requiring further research. Few participants explicitly described their reflective practices. However, reflection on practice through “structured critical reflection and analysis” is an important and crucial component of learning and development [28, p. 264]. Research which explicatively investigates how medical teachers use reflection and reflective practice as an aid to learning to teach and what that practice looks like may expand existing literature [41–45]. Secondly, studies which specifically investigate how medical teachers receive feedback, and how that feedback benefits development of their teaching practice may contribute to the apparently limited literature concerning feedback and workplace learning.

Thirdly, organizational structures, including cultural differences across specialties and sites of training, have the potential to inhibit medical teaching and learning opportunities. More research in this area may identify characteristics of supportive learning communities [28]. Lastly, lack of self-awareness of the need to improve teaching may be a significant barrier to learning [11]. Research which explicatively investigates medical teachers’ self-awareness of teaching skills may identify individual traits and/or organizational structures that contribute to heightened self-awareness of teaching skills.

## Conclusion

Medical education requires an increasing number of appropriately trained and experienced medical teachers. This study sought to add to and fill a gap in the existing literature by exploring how medical teachers perceived teaching, and how they described learning and development of teaching medical trainees. The findings may have implications for faculty development programs as they suggest that the participants’ valued informal teaching practices, and their predominant resource for learning how to teach was informal experiential learning, both in and out of the workplace. Further research which investigates medical teachers’ reflective practice, feedback opportunities, supportive organizational structures, and self-awareness of teaching skills may contribute to their learning needs and provide additional insights into faculty development needs.
